# A Frameshift Mutation in *KIT* is Associated with White Spotting in the Arabian Camel

**DOI:** 10.3390/genes8030102

**Published:** 2017-03-09

**Authors:** Heather Holl, Ramiro Isaza, Yasmin Mohamoud, Ayeda Ahmed, Faisal Almathen, Cherifi Youcef, Semir Gaouar, Douglas F. Antczak, Samantha Brooks

**Affiliations:** 1 Department of Animal Sciences, UF Genomics Institute, University of Florida, Gainesville, FL 32610, USA; heather.holl@ufl.edu; 2 Department of Small Animal Clinical Sciences, College of Veterinary Medicine, University of Florida, Gainesville, FL 32608, USA; isazar@ufl.edu; 3 Department of Genetic Medicine, Weill Cornell Medical College in Qatar, Doha, Qatar; yam2012@qatar-med.cornell.edu (Y.M.); ays2003@qatar-med.cornell.edu (A.A.); 4 Veterinary Public Health and Animal Husbandry, College of Veterinary Medicine and Animal Resources, King Faisal University, Al-Ahsa 31982, Saudi Arabia; faisalvet@hotmail.com; 5 Department of Biology, University of Abou Bekr Belkaïd, Tlemcen 13000, Algeria; cherifi.youcef@ymail.com (C.Y.); suheilgaouar@gmail.com (S.G.); 6 Baker Institute for Animal Health, College of Veterinary Medicine, Cornell University, Ithaca, NY 14853, USA; dfa1@cornell.edu

**Keywords:** Arabian camel, dromedary camel, white spotting, *KIT*

## Abstract

While the typical Arabian camel is characterized by a single colored coat, there are rare populations with white spotting patterns. White spotting coat patterns are found in virtually all domesticated species, but are rare in wild species. Theories suggest that white spotting is linked to the domestication process, and is occasionally associated with health disorders. Though mutations have been found in a diverse array of species, fewer than 30 genes have been associated with spotting patterns, thus providing a key set of candidate genes for the Arabian camel. We obtained 26 spotted camels and 24 solid controls for candidate gene analysis. One spotted and eight solid camels were whole genome sequenced as part of a separate project. The spotted camel was heterozygous for a frameshift deletion in *KIT* (c.1842delG, named *KIT^W1^* for *White spotting 1*), whereas all other camels were wild-type (*KIT^+^*/*KIT^+^*). No additional mutations unique to the spotted camel were detected in the *EDNRB*, *EDN3*, *SOX10*, *KITLG*, *PDGFRA*, *MITF*, and *PAX3* candidate white spotting genes. Sanger sequencing of the study population identified an additional five *KIT^W1^*/*KIT^+^* spotted camels. The frameshift results in a premature stop codon five amino acids downstream, thus terminating KIT at the tyrosine kinase domain. An additional 13 spotted camels tested *KIT^+^*/*KIT^+^*, but due to phenotypic differences when compared to the *KIT^W1^*/*KIT^+^* camels, they likely represent an independent mutation. Our study suggests that there are at least two causes of white spotting in the Arabian camel, the newly described *KIT^W1^* allele and an uncharacterized mutation.

## 1. Introduction

Coat color variation was valued throughout animal domestication [1]. Human selection favors unique morphological characteristics, resulting in the diverse array of coat pigmentation present in many species. Many genes are associated with white spotting patterns in domestic animals [2]. However, color variation is often associated with other behavioral or health traits, due to the phenomenon of genetic pleiotropy [3]. White spotting in particular is linked with conditions such as anemia, deafness, gastrointestinal abnormalities, skin cancers, and sterility [4]. However, while white spotting phenotypes are common among domesticated species, not all have known pleiotropy.

The Arabian camel is an important domestic species selected for draught, meat, milk, racing, and riding. Though usually with a uniform coat of varying shades of brown or black, there are rare spotted or fully white populations [5,6,7,8]. To our knowledge, there have been no published studies on the genetics of camel coat color patterns. In this work, we report on a candidate gene approach using whole genome sequencing to characterize the genetics of white spotting.

## 2. Materials and Methods

### 2.1. Sample Collection

Sample collection for this study was approved by the University of Florida Institutional Animal Care and Use Committee (IACUC) under protocol #201408506. All animals were voluntarily enrolled by private owners. Blood or hair samples were collected from 50 Arabian camels. Photographs were obtained for all but three animals, which were used to assess phenotype (Table 1). For the three animals without photos, phenotype was recorded as a written description at the time of sample collection. Individuals were identified as spotted if they exhibited a region of white fur with pink skin underneath on a background of normal pigmentation. Pedigree information was only available for five dam-offspring pairs (Table 1). Additionally, camels US12 and US15 as well as US14 and US17 were identified as possible half siblings, though the owner was not certain. DNA was extracted using either the Gentra Puregene buffy coat protocol (Qiagen, Valencia, CA, USA) or a modified Puregene protocol for hair [9].

### 2.2. Next-Generation Sequencing

Whole genome sequencing was performed on eight camels (QA1-5, US1, US3-4, US6). Each sample was sequenced on a single lane of an Illumina HiSeq 2500 with 2 × 100 bp reads. We obtained an average of 184 million read pairs per lane. Raw sequencing reads were submitted to the ENA under accession number PRJEB15365. A custom reference sequence file was prepared, comprised of Arabian camel scaffolds matching known white spotting genes *EDNRB*, *EDN3*, *SOX10*, *KIT*, *KITLG*, *PDGFRA*, *MITF*, and *PAX3* [10,11] (Supplementary Table S1). Sequencing reads were aligned with BWA 0.7.12-r1039 using default parameters, and were converted to BAM format using SAMtools 0.1.19-44428cd [12,13].

Predicted dromedary camel mRNA sequences for the candidate genes were obtained from Genbank and aligned to the custom reference using BLAT 20140318 [14] (Supplementary Table S1). Alignments were visualized in IGV 2.3.55 [15]. The coding regions and immediately surrounding introns were manually inspected for variant annotation (Supplementary Table S2). At least three reads for homozygous variants and six for heterozygous variants were required to record a genotype.

### 2.3. Variant Validation and Genotyping

Variants observed only in the spotted camel genome were selected for further evaluation. PCR primers were designed using the reference scaffolds and Primer3 [16] (Supplementary Table S3). Additional samples were genotyped by Sanger sequencing of PCR amplicons. Chromatograms in PDF format were visually inspected to assess genotype. Sanger sequences for camel *KIT* exons 12 and 13 were submitted to Genbank and are available under accession numbers KX784929 (*KIT^W1^*) and KX784928 (*KIT^+^*).

## 3. Results and Discussion

**Figure 1:**
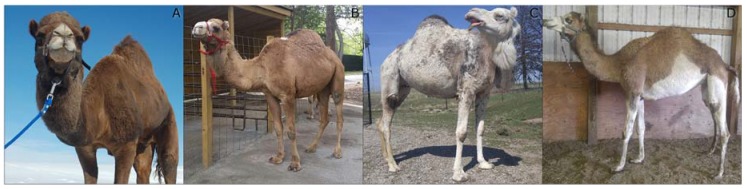
Range of white spotting phenotypes in this study. (**A**) A minimally spotted camel (US30), displaying white patches on both upper lips. (**B**) A minimally spotted camel (US25), showing a facial marking and white spot in front of the hump. (**C**) An extensively spotted “mottled white” camel (US28), with pigmented regions concentrated along the topline. (**D**) A moderately spotted “clear white” camel (US34), showing clear definition between pigmented and white regions.

The degree of white spotting of each animal varied greatly, ranging from white only on the lips, to white on approximately 80% of the body (Table 1, Figure 1). Camels with extensive markings had blue eyes. Samples US27 and US41 were recorded as solid white as there were no obvious colored patches of fur. “Mottled white” indicates that the border between the colored and white fur was jagged and uneven, and/or with regions where colored and white fur were intermingled (Figure 1C). “Clear white” indicates a well-defined border between colored and white fur, though with possible flecks of color within predominantly white patches (Figure 1D). Minimally spotted camels often had white restricted to the “points”—the face, legs, and tail. The US spotted camels were visibly smaller in overall body size than the solid camels, though no quantitative measures were assessed. Two of the owners mentioned that spotted camels are known for being roughly 30 cm shorter at maturity.

Most US owners did not have information on the colors of the parents of the spotted camels. One owner described that many of the breeders he knew of bred white spotted bulls to non-spotted cows in order to produce color. He knew of one spotted bull (~50% of the body with white spotting) that produced all minimally spotted offspring (dark eyes with white on the face, legs, and/or tail) out of solid cows. However, the mottled white phenotype is not likely to be incompletely dominant, as one of the extensively marked camels in the study (US28) produced a completely non-spotted calf (US29). We thus assumed the mottled white trait would be dominant based on similarities to white spotting patterns in the horse.

The spotted Algerian camels, known locally as Azerghaf or Zarwala, are found in the southwest regions of the country. Some tribes prefer the spotted camels as they are born deaf, and thus are easier to raise. Interviews with local farmers indicate the color is likely autosomal recessive in inheritance. A secondary type of spotted Algerian camel (characterized by a white spot on the forehead, found in the extreme south) was not available for genotyping.

**Figure 2:**
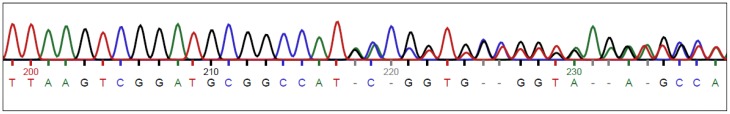
Sanger sequencing confirms the presence of a heterozygous deletion. The *KIT*:c.1842delG variant results in a frameshift five bases downstream, truncating the protein at the intracellular tyrosine kinase domain.

Variant screening identified one mutation in *KIT* as a likely candidate (Supplementary Table S2). There were no variants detected in *PAX3*, whereas candidate genes *SOX10, MITF,* and *KITLG* only had variants in the untranslated regions (only one of which was uniquely present in spotted camel US4). Silent variants in *KIT*, *EDN3*, *EDNRB*, and *PDGFRA* were found in both US4 and non-spotted camels (QA1-5, US1, US3, US6). There was one homozygous missense variant in *PDGFRA* present in US4, but it was not considered further as QA4 was heterozygous for this variant as well. The only coding variant unique to US4 was a heterozygous single base deletion within exon 12 of 21 in *KIT* (c.1842delG). This mutation results in a frameshift, leading to a premature stop codon five amino acids downstream (p.M614IfsX5). This mutation ultimately truncates the protein at the intracellular tyrosine kinase domain. Sanger sequencing identified eight additional heterozygotes and no homozygous mutants (Figure 2, Table 2). Five heterozygotes were in the mottled white category, whereas the remaining four animals had too little white to define as clear or mottled (Table 1). Based on the appearance of the minimally spotted camels, we have termed the allele *KIT^W1^* for *White spotting 1*.

*KIT*, *v-kit Hardy-Zuckerman 4 feline sarcoma viral oncogene homolo*g, is one of the most commonly implicated genes for white spotting phenotypes in animals [17,18,19,20,21,22,23,24]. There are four reported frameshift mutations in the domestic horse, all of which were associated with extensive white spotting [17,25,26]. *KIT* encodes a tyrosine kinase receptor involved in the development of erthyrocytes, melanocytes, germ cells, mast cells, and interstitial cells of Cajal [27]. As a result, loss-of-function mutations have been associated with a variety of negative pleiotropic effects, and in some cases are hypothesized to be homozygous lethal [20,28,29]. However, not all *KIT* variants have reported pleiotropy, and specific variants in cats and horses were shown to have normal hematological parameters [18,30]. All nine camels with the *KIT*:c.1842delG variant were mature without obvious health issues, although detailed histories were not available. Additionally, one of the animals had two previous successful pregnancies, and thus was fertile. The *KIT^W1^* allele does not appear to be detrimental in heterozygotes, although homozygous embryos are likely not viable, similar to dogs with a frameshift mutation in *KIT* [20].

The appearance of the nine *KIT^W1^/KIT^W+^* camels was highly variable. Minimally spotted animals (white on face, legs, or tail only) had dark eyes, whereas the more extensively marked camels had blue eyes (Table 1). Blue eyes are commonly associated with white markings in a variety of species. Several of the equine *KIT* variants also show considerable variation in the degree of spotting, including horses with white found only on the head and legs [25,31,32]. Intense selection for the degree of white spotting in the mouse has demonstrated that a large number of additive and modifying loci cumulatively contribute to overall color, in addition to non-genetic factors [33]. Heterozygous *KIT* mice display the full range of color, from completely solid to completely white. It is thus likely that the more extensively marked mottled white camels possessed additional genetic variants that we did not detect.

As the remaining spotted camels were wild-type for *KIT*:c.1842delG, they likely have a different mutation responsible for white spotting. The overall appearance of the moderately marked animals was quite distinct between the “mottled white” and “clear white” patterns, with the “clear white” animals appearing similar to the “splashed white” coat color of the horse [34]. Interestingly, both the Algerian spotted camels and some splashed white horses have a congenital deafness. Splashed white is associated with mutations in *MITF* and *PAX3*, both of which are genes implicated in the human Waardenburg Syndrome (characterized by white patches of skin, blue eyes, and some degree of deafness). Thus, *MITF* and *PAX3* are attractive candidates for the “clear white” Arabian camel phenotype.

## 4. Conclusions

In conclusion, the c.1842delG mutation in *KIT* is likely responsible for one form of white spotting in the dromedary camel. We propose to name this allele *KIT^W1^* for *White spotting 1*. However, due to the wide variation in *KIT^W1^*/*KIT^+^* animals, and the presence of *KIT^+^*/*KIT^+^* spotted camels, there are likely other mutations that we did not characterize. Further research should elucidate the genes responsible for other white phenotypes.

## Figures and Tables

**Table 1:** Origin and coat color information for the camels in this study.

ID	Origin	Coat	Eyes	Markings	Genotype
QA1	Qatar	Solid Brown	Dark	None	*KIT^+^*/*KIT^+^*
QA2	Qatar	Solid Brown	Dark	None	*KIT^+^*/*KIT^+^*
QA3	Qatar	Solid Brown	Dark	None	*KIT^+^*/*KIT^+^*
QA4	Qatar	Solid Brown	Dark	None	*KIT^+^*/*KIT^+^*
QA5	Qatar	Solid Brown	Dark	None	*KIT^+^*/*KIT^+^*
US1	US Farm 1	Solid Brown	Dark	None	*KIT^+^*/*KIT^+^*
US3	US Farm 1	Solid Brown	Dark	None	*KIT^+^*/*KIT^+^*
US4	US Farm 2	Spotted Brown	Blue	Mottled white throughout body/neck/head, colored topline	*KIT^W1^*/*KIT^+^*
US5	US Farm 2	Spotted Brown	Blue	Mottled white on head and lower half of body, dark brown patch on rump	*KIT^W1^*/*KIT^+^*
US6	US Farm 3	Solid Brown	Dark	None	*KIT^+^*/*KIT^+^*
US12 ^1^	US Farm 4	Spotted Brown	Blue	Clear white legs/belly, mostly white face	*KIT^+^*/*KIT^+^*
US13	US Farm 4	Spotted Brown	Blue	Clear white legs/belly, half white face	*KIT^+^*/*KIT^+^*
US14 ^2^	US Farm 4	Spotted Brown	Dark	Mottled white to knees/hocks, wide stripe on face, white tail tip	*KIT^W1^*/*KIT^+^*
US15 ^1^	US Farm 4	Spotted Brown	Blue	Clear white from legs to flanks, mostly white face	*KIT^+^*/*KIT^+^*
US16	US Farm 4	Spotted Brown	Blue	Clear white to stifle/knee, clear white belly spot, white nose	*KIT^+^*/*KIT^+^*
US17 ^2^	US Farm 4	Spotted Brown	Dark	Mottled white to knees/hocks, white spot on inner hindquarters, white tail tip	*KIT^W1^*/*KIT^+^*
US25	US Farm 5	Spotted Brown	Dark	Wide stripe on face, white spot on withers, white toe, white tail tip	*KIT^W1^*/*KIT^+^*
US26 ^3^	US Farm 5	Solid Brown	Blue	Light roaning	*KIT^+^*/*KIT^+^*
US27 ^3^	US Farm 5	Solid White	Part-Blue	None	*KIT^+^*/*KIT^+^*
US28 ^4^	US Farm 5	Spotted Brown	Blue	Mottled white throughout body/neck/head, colored topline	*KIT^W1^*/*KIT^+^*
US29 ^4^	US Farm 5	Solid Brown	Dark	None	*KIT^+^*/*KIT^+^*
US30	US Farm 5	Spotted Brown	Dark	White lips, light roaning	*KIT^+^*/*KIT^+^*
US31	US Farm 6	Spotted Brown	Dark	Wide stripe on face	*KIT^W1^*/*KIT^+^*
US32	US Farm 6	Solid Brown	Dark	None	*KIT^+^*/*KIT^+^*
US33	US Farm 6	Spotted Brown	Dark	White to elbows, white tail tip	*KIT^W1^*/*KIT^+^*
US34	US Farm 6	Spotted Brown	Blue	Clear white to flank, half white face	*KIT^+^*/*KIT^+^*
US35	US Farm 6	Solid Brown	Dark	None	*KIT^+^*/*KIT^+^*
US36	US Farm 6	Spotted Brown	Blue	Clear white to flank, half white face	*KIT^+^*/*KIT^+^*
US37	US Farm 6	Solid Brown	Dark	None	*KIT^+^*/*KIT^+^*
US38	US Farm 6	Solid Brown	Dark	None	*KIT^+^*/*KIT^+^*
US39	US Farm 6	Solid Brown	Dark	None	*KIT^+^*/*KIT^+^*
US40	US Farm 6	Solid Brown	Dark	None	*KIT^+^*/*KIT^+^*
US41	US Farm 6	Solid White	Dark	None	*KIT^+^*/*KIT^+^*
US42 ^5^	US Farm 6	Solid Brown	Dark	None	*KIT^+^*/*KIT^+^*
US43 ^5^	US Farm 6	Spotted Brown	Blue	Clear white to knees, white nose	*KIT^+^*/*KIT^+^*
US44 ^6^	US Farm 6	Solid Brown	Dark	None	*KIT^+^*/*KIT^+^*
US45 ^6^	US Farm 6	Solid Brown	Dark	None	*KIT^+^*/*KIT^+^*
US46 ^7^	US Farm 6	Solid Brown	Dark	None	*KIT^+^*/*KIT^+^*
US47 ^7^	US Farm 6	Solid Brown	Dark	None	*KIT^+^*/*KIT^+^*
US48	US Farm 6	Spotted Brown	Dark	White stripe on face, white tail	*KIT^W1^*/*KIT^+^*
US49	US Farm 6	Spotted Brown	Dark	White tail tip	*KIT^+^*/*KIT^+^*
US50	US Farm 6	Solid Brown	Dark	None	*KIT^+^*/*KIT^+^*
SA1	Algeria	Spotted Brown	Blue	Clear white belly to flank, white face	*KIT^+^*/*KIT^+^*
SA2	Algeria	Spotted Brown	Blue	White to knees, white face	*KIT^+^*/*KIT^+^*
SA3	Algeria	Spotted Brown	Blue	Clear white to flanks, mostly white face	*KIT^+^*/*KIT^+^*
SA4	Algeria	Spotted Brown	Blue	Clear white to flanks, mostly white face	*KIT^+^*/*KIT^+^*
SA5	Algeria	Spotted Brown	Blue	White to knees, half white face	*KIT^+^*/*KIT^+^*
SA6	Algeria	Spotted Black	Blue	No photo available	*KIT^+^*/*KIT^+^*
SA7	Algeria	Spotted Black	Blue	No photo available	*KIT^+^*/*KIT^+^*
SA8	Algeria	Spotted Black	Blue	No photo available	*KIT^+^*/*KIT^+^*

^1^ Possible half sibling pair; ^2^ Possible half sibling pair; ^3^ Dam (US26) offspring (US27) pair; ^4^ Dam (US28) offspring (US29) pair; ^5^ Dam (US42) offspring (US43) pair; ^6^ Dam (US44) offspring (US45) pair; ^7^ Dam (US46) offspring (US47) pair.

**Table 2:** Genotyping results for the *KIT*:c.1842delG mutation (*KIT^W1^*).

Phenotype	WT/WT	WT/del	del/del	Total
Solid Brown	22	0	0	22
Solid White	2	0	0	2
Clear White	10	0	0	10
Mottled White	0	5	0	5
Other Spotted	7	4	0	11
Total	41	9	0	50

## Supplementary Materials

The following are available online at www.mdpi.com/2073-4425/8/3/102/s1, Table S1: NCBI accession numbers for the scaffolds and proteins used as reference sequences, Table S2: Detected variants in candidate white spotting genes, Table S3: PCR primers used for Sanger sequencing validation.
